# The Use of Low-Cost Unmanned Aerial Vehicles in the Process of Building Models for Cultural Tourism, 3D Web and Augmented/Mixed Reality Applications

**DOI:** 10.3390/s20195457

**Published:** 2020-09-23

**Authors:** Tomasz Templin, Dariusz Popielarczyk

**Affiliations:** Department of Geodesy, Institute of Geodesy and Civil Engineering, Faculty of Geoengineering, University of Warmia and Mazury in Olsztyn, 10–719 Olsztyn, Poland; dariusz.popielarczyk@uwm.edu.pl

**Keywords:** UAV/UAS, smart city, smart tourism, tourism services, cultural heritage, AR, MR, 3D Web

## Abstract

Unmanned Aerial Systems (UAS) are widely used in low-cost photogrammetry. Even small Unmanned Aerial Vehicles (UAV) can deliver valuable data for the inventory of inaccessible and dangerous areas or objects. The acquisition of data for 3D object modeling is a complicated, time-consuming, and cost-intensive process. It requires the use of expensive equipment and often manual work as well as professional software. These are major barriers limiting the development of modern tourist platforms that promote local attractions. Information technologies offer new opportunities for the development of the services market, including the development of smart tourism services, as an integral part of the smart city concept. 3D models are an important element of this process as they form the basis for the use of new visualization technologies, such as Virtual, Mixed, and Augmented Reality (VR/MR/AR). 3D modeling provides a new opportunity to use AR/MR technology to present information about objects, virtual tours of the historic buildings, and their promotion. It also creates an opportunity to preserve the architectural heritage and preventive maintenance of buildings. Despite the increasing use of new measuring platforms and computer modeling techniques, the implementation of 3D building models in smart tourism services is still limited, focusing more on the results of scientific projects rather than on the implementation of the new ones. The paper presents an universal methodology for the inventory of historical buildings using low-cost UAVs. It describes the most important aspects related to the process of planning UAV measurement missions and photogrammetric data acquisition. The construction of 3D models and the possibilities of their further use to build smart tourism services based on Web/AR/MR/VR technology was also presented.

## 1. Introduction

Intelligent solutions are the foundation for creating platforms that integrate people, society, and businesses. They are a basis for building environmentally-friendly smart platforms that have a strong positive impact on human life. Smart communities make the lives of citizens better by supporting infrastructure needs, reimagining cultural awareness, and eliminating social inequality. The vision of smart cities has become a common trend in the development of modern towns and metropolises [1]. Nowadays, it is also the subject of many scientific studies and commercial projects. A recent review of solutions implemented at the local, regional, or municipal government level was presented by Camero et al. [2]. 

There are new and innovative ways to share and communicate data, exchange views, and collaborate to create better, safer, and more sustainable smart communities. The concept of "smart" is based on the intensive deployment of information and communication technology (ICT) infrastructure, as well as on the proliferation of mobile technology and its applications [3]. Smart platforms and modern visualization technology allow for the synergy of integration, innovation, and growth [4]. 

Information technologies have offered new possibilities for the development of the market of services [5]. Smart cultural heritage services are one of the possible ways of unlocking the potential of these technologies. They offer access to historical objects and preserve cultural heritage and the arts. The term has been defined by Borda [6] as services that focus on collaborative approaches, making cultural data open and freely available. They involve consumers in the process of experiencing the content they are discovering, providing access to cultural objects and experiences also across distance. In the literature several authors have presented example of such services [7,8,9,10].

In the literature, there are many definitions of smart tourism and its role in smart cities [11]. One of the classical approaches defines smart tourism as a subset of the smart city concept, aiming to provide tourists with solutions that address specific travel-related needs [12]. It specifically highlights smart tourism as an integral component of the overall systems and processes. 

Cultural heritage tourism involves visiting places that are significant to the past or present cultural identity of a particular group of people [13]. The high availability of mobile apps has led to significant changes in the way people get information, consume digital content, and spend free time [14]. This is particularly evident in the young generation, for whom the participation of mobile devices in this process is becoming obvious. The results of many studies have confirmed that people want to decide when and where they want to access content and how to share it [15]. This is a big chance to promote local historical attractions.

In the world of cultural heritage, an increasing number of applications are outdoor context-aware, location-based services (LBS) [16]. They use modern visualization methods, especially augmented reality (AR), mixed reality (MR) and virtual reality (VR) [17]. The most popular mobile applications are created for museums, galleries and for promotion of historical events [18,19,20]. 

Rapidly developing technologies allow the provision of building sophisticated services with an increasing number of features. However, they are affected by the risks associated with the complexity of the system and difficulties in meeting the requirements [21]. Another significant problem is the lack of availability of reliable, up-to-date digital content specific for cultural heritage. 

Modern laser scanning methods and photogrammetric techniques allow for quick acquisition of 3D scene data [22]. Technological progress has led to the creation of an increasing number of object and environment models [23]. Nevertheless, the process of creating advanced 3D models is expensive, time consuming and requires manual work. This means that research is usually conducted in the most prestigious, attractive historical locations for famous historical objects.

Unmanned Aerial Systems (UAS) are widely used in low-cost photogrammetry [24,25,26,27,28]. Even small aerial vehicles (AVs) can provide valuable data for the inventory of inaccessible and dangerous areas or objects. The UAS multi-sensor approach to 3D modeling and reconstruction of cultural heritage objects has been studied by many authors [29]. The combination of UAS with laser scanning can provide excellent fast data acquisition capabilities, also for AR/MR purposes [30].

Recently, the scientific community has been working on Historic Building Information Modelling (HBIM) to improve the interoperability between information/data in various types and formats [31]. BIM is an interdisciplinary platform connecting specialists from various fields. It is a centralized access point that helps many users work simultaneously. The multidimensional character of the presented models allows for a holistic overview of the object considering all its aspects, from the design to demolition. Based on the literature, BIM is a fully seven-dimensional (7D) platform. The first three dimensions refer to geometry, the 4D dimension adds dynamics by temporal simulation, the 5D includes costs, the 6D is related to building life cycle management, and the 7D concerns facility and asset management [32].

BIM is a complete solution that meets the expectations of professional users. It provides an interface for sharing information throughout the whole building life cycle. Currently, several commercial applications are available in the market. They offer advanced algorithms for creating, analyzing, and further visualizing building models [33]. There are many studies in the literature concerning the use of VR/AR/MR for the visualization of historical buildings [34]. However, they show the need to develop solutions that allow the use of 3D products also for cultural tourism and services for individual customers.

The challenge in the field of cultural heritage services is the lack of universal, easy to implement standards for preparation of digital content for Web/AR/MR/VR services. The existing solutions are usually the result of complex research projects, focusing on individual objects in dispersed locations. They often require advanced computer hardware, expensive software, or have limited spatial coverage. 

The article presents the methodology of using UAV data for historical objects to prepare digital 3D assets for Web3D, AR/MR/VR smart cultural heritage services. The raw data is processed and used in GIS applications and optimized for typical tourist services that are based on Web/AR/MR/VR technology. The proposed method indicates the possibility of using low-cost UAV for fast data acquisition in the process of building 3D models of historical buildings. The following sections propose the phase-in of planning measurements and data acquisition. They also describe the problems with the implementation of the UAV mission. Laser scanner measurements and their development are also presented. Then the process of creating the 3D building model and its optimization for mixed and augmented reality solutions is shown. The visualization of the building model is in itself accessible through web browsers as a 3D model, a mobile application as augmented reality content, a windows application with mixed reality; alternatively, it can be experienced as a 3D immersion in virtual reality (VR).

## 2. Materials and Methods

### 2.1. Problem

Historic buildings are an important part of cultural property. They have their history and the look of buildings has evolved throughout the ages. Only the buildings which have been highly valued, both by rulers and citizens, have survived the wear of time. Usually these are buildings that fulfilled an important religious role in the society, e.g. churches, cathedrals and temples [35]. Recently another type of building has been added to this list: industrial and military heritage. 

Small towns or cities have tight budgets for the preservation of historical buildings. There is a shortage of funds to promote these places and their history and to build tourist services that encourage people to visit them. It caused many cultural heritage locations to be hidden from tourists, especially in countries with many historical attractions. Tourists tend to consider only the most popular towns, turning their back on other beautiful places. 

Despite the increasing use of new measuring platforms and computer modeling techniques, the implementation of 3D building models in smart tourism services is still limited, focusing more on the results of scientific projects rather than on the implementation of the new ones. In the literature on the subject, few studies concentrate on the need for centralized 3D cultural heritage archives [23,36]. Most of them focus on peer-reviewed 3D models and a methodological approach useful for producing 3D digital content of cultural heritage objects in the context of digital archives.

Computer-generated three-dimensional models are the foundation of modern smart tourism services. They can be used in hand-held devices to promote the region and present useful information not only to professional users, specialists in architecture, civil engineering, cultural heritage documentation, and preservation. It could be a unified platform for a wide user community interested in cultural tourism. Digital representations (DRs) supported by modern visualization techniques (VR/AR/MR) can fulfill these visions effectively, revealing the 3D digital content. The overlaid content can be derived from the existing data sets or it can be obtained from modern measurement sensors. One of the cost-effective sources of 3D building models is UAV/UAS.

The following subchapters present a method that uses low-cost UAV to quickly and efficiently acquire data on a historical water tower located in the Modlin fortress in Poland. At the beginning we introduce HBIM systems and typical workflow processing. The process of building HBIM is complex, interdisciplinary and requires standards and cooperation of specialists from different fields. The construction of the 3D model is the stage that requires the selection of appropriate measurement technologies and maintenance of accuracy standards. 

In the next step we present the modelling methodology for smart tourism services. The process of creating 3D models of buildings is shown. The model built with the use of the proposed methodology after the accuracy analysis can be used directly in the process of building HBIM, or indirectly among others, for planning the measurement works, visual assessment of the building’s condition or planning renovation works. 

In the following subchapters we present the historical model of the building and its further optimization for mixed and augmented reality purposes. Based on the collected data, we make different versions of a 3D textural building model. In the end, the visualization of building models themselves is accessible through web browsers as a 3D model, a mobile application as augmented reality content, and a windows application with mixed reality. The tower visualization can also alternatively be experienced as 3D immersion in virtual reality (VR).

### 2.2. Typical Processing Workflow for HBIM

**Figure 1:**
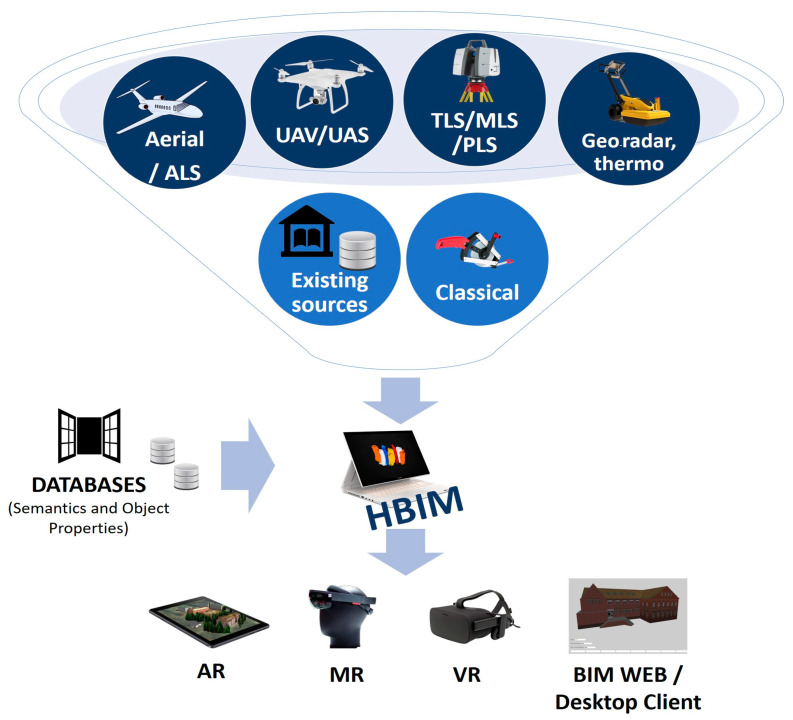
A typical methodology for implementation of an HBIM.

An unprecedented progress of technologies caused the emergence of many new measurement platforms and sensors. They are able to quickly obtain comprehensive data on historic buildings. They allow us to increase the speed of data acquisition, improve the level of detail and spatial resolution. The development of measurement technologies has also increased the amount of collected data, added new attributes, and additional parameters describing material properties. A comprehensive review of the measurement methods used for the geometric documentation of cultural heritage was presented by Georgopoulos [37]. A typical methodology for implementation of an HBIM is shown in Figure 1. 

Regarding the measurement platform, the following sources of data can be specified:

- Existing materials, drawings, photographs, both in digital and analog form (existing sources),
- Traditional measuring methods (geodetic, other),
- Photogrammetric imagery and aerial laser scanning,
- Data from platforms such as UAV/UAS (passive and active sensors),
- Laser scanning using a terrestrial, mobile, and personal platform,
- Product of non-destructive tests (geo-radar, thermography, sonic tests),
- Other methods, including minor destructive and destructive tests.

New sensors types, including optical, acoustic, laser scanning, radar, thermal devices provide the opportunity to build complex models with unprecedented levels of detail. Depending on the purpose and requirements, different methodologies can be used. Digital documentation of cultural heritage consists of three stages:

- Acquiring raw data,
- Processing raw data,
- Generating the model.

Data acquisition is an important step in the modeling process. This is the first stage at which data is usually acquired from multiple sources. Several pre-processing steps should be carried out on the raw data before modeling. These steps include registration, segmentation/filtering, triangulation, texturing, and orthophoto creation. Typically, integration of data from multiple data sources is required.

The modeling phase is the last and most challenging stage. The literature presents several different strategies for building BIM models [38,39,40]. Two main working methods used to build 3D models are direct and parametric modelling [41]. Some authors also propose the third one as a hybrid solution [42].

The direct modeling process uses the point cloud as the basis for vectoring the profiles required to construct a 3D model. In the parametric method, architectural shapes are recognized and segmented based on raw data. The conversion process can be performed by manual, semi-automatic or automatic modelling [43]. All recognized objects are added to HBIM databases as surfaces (planes, curves, or extrusion), volumes, and complex objects.

At this stage, pre-designed libraries of basic architectural and structural elements are often used. Due to the character of existing and historic buildings, the extensive libraries of predefined parametric objects contained in BIM software do not always contain all the necessary objects and commands for modeling irregular shapes. However, there is great potential for improvement in the modeling process with the development of new parametric libraries dedicated to existing and historic buildings. The results of this work are presented, among others, in the following studies [44,45,46,47].

With the technological progress we have new algorithms that automate the process of point cloud analysis and object recognition. Automation gives the possibility to shorten time and thus reduce costs of BIM projects. There is a lot of work being performed at the moment. The process of automatic object recognition and object detection gives promising results, but does not automate the entire process from point cloud to BIM. A comprehensive overview of current solutions can be found in Dore et al. [48]. Usually, the construction process is conducted using standard BIM software packages (e.g., Revit, ArchiCAD, OpenBuildings Designer, Teckla) or specialized applications such as the Rhinoceros software.

### 2.3. Modeling for Smart Tourism Services Based on UAV Data

Today the preferred surveying technique for the 3D documentation of historic buildings is terrestrial laser scanning. High-speed, high-accuracy, and dedicated processing software enable efficient creation of 2D, 3D, and BIM models. However, the high price and limitations relating to measuring high objects (roofs of buildings), sometimes renders their use to be limited or requires additional equipment.

UAVs combined with RGB and multispectral cameras represent platforms to collect high-quality spatio-temporal data, including digital surface models (DSMs) and orthorectified images (orthophotos) or 3D models of objects. Even small UAVs can deliver valuable data for the inventory of inaccessible and dangerous areas or objects.

**Figure 2:**
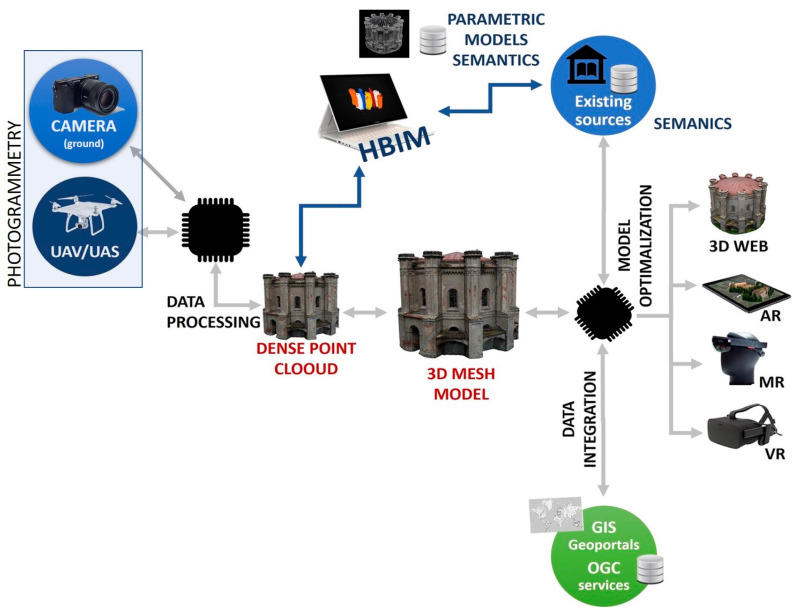
Review of the methodology adopted for the reconstruction of 3D buildings using UAV/UAS.

Figure 2 presents the proposed methodology that describes the use of UAVs and ground images from Digital Single Lens Reflex (DSLR) cameras for preparing a 3D textured model for AR/VR purposes. Typically, after processing the UAV/DSLR raw data dense point cloud is generated. Based on this cloud, wireframes are generated and then using parametric modeling or NURBS curves, BIM models are created. In the proposed approach, we suggest unstructured geometric reconstruction of the external surface of the building based on point cloud. The created model is then optimized in subsequent steps for use in AR and VR mobile applications.

### 2.4. Study Area

**Figure 3:**
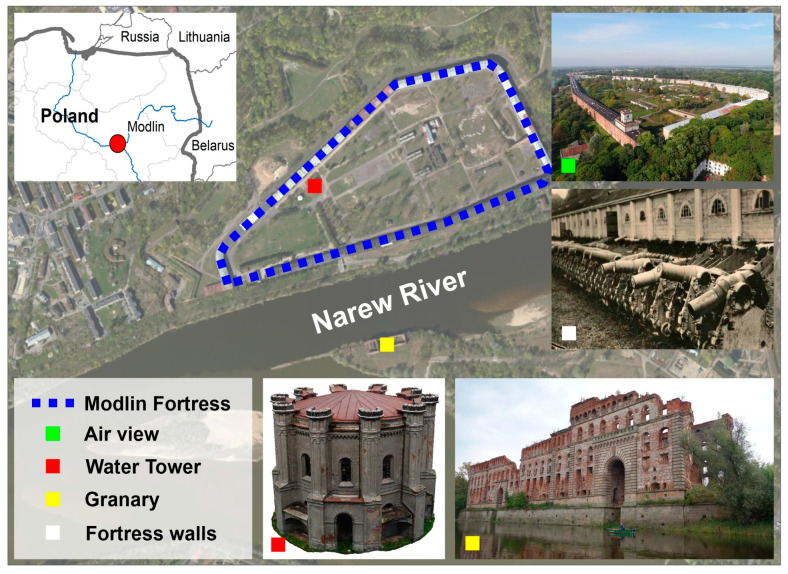
Study area.

The Modlin fortress is one of the largest 19th century fortresses in Poland. The fortress is located on the territory of Nowy Dwór Mazowiecki, at the fork of the Vistula, Narew and Wkra rivers (Figure 3). It is situated 34 km north-west of the Polish capital, Warsaw. It consists of a citadel on the right bank of the Narew River, fortified forecourts—Kazunski and Nowy Dwór—and two rings of forts. The fortress was initially built by the French in the years 1806–1812. The main barrack located within the complex is the longest building in Europe, with a length of 2250 m. It can accommodate up to 20,000 soldiers.

**Figure 4:**
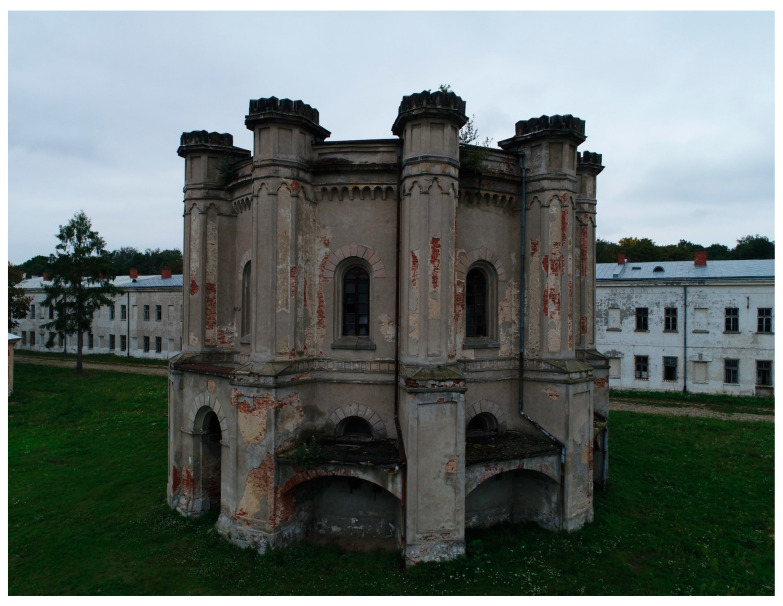
The water tower inside Modlin fortress—recent look.

Inside the barracks, the courtyard features a beautifully designed neo-Gothic water tower (Figure 4). The water tower was built around 1847 on a circle plan (octagon). A huge water reservoir placed on the first floor supplied the barracks with water. The water was drawn by means of spouts placed outside the building (in special, roofed recesses). Each was placed in the open mouth of a lion. In the walls of the recesses, there were stone troughs for watering horses. The water tower was used in the interwar period (until 1939) for watering horses. 

Today the tower is no longer used. Although no maintenance work has been carried out on it for years, it is still an ornament of the barracks courtyard (it resembles a palace in its external appearance). It is a unique tourist facility. The building can only be seen with a guide because it is located in the middle of the closed barracks courtyard, which is private property.

Today, the water tower is one of the tourist attractions inside Modlin fortress. Since its first construction in 1847 it has been demolished and rebuilt several times. In addition to suffering human devastation, forces of nature have caused the building to decay. The original shape of the building, it’s size and height, makes it a challenging restoration project. UAV surveys allow accurate assessment of the damage, stability of the structure and required restoration works.

## 3. Measurements

The water tower building inventory at the Modlin Fortress was performed with two independent measuring systems: an UAS and a terrestrial laser scanner (TLS).

### 3.1. UAV Data Acquisition

One of the most important steps in using an UAV to make a 3D model is to choose the right moment to fly. In addition to avoiding strong winds and rain, it is also important to choose a little cloudy day around noon, when the shadows are not too strong nor too long. During our research we chose an overcast day and the photos were taken between 1 and 3 pm.

**Figure 5:**
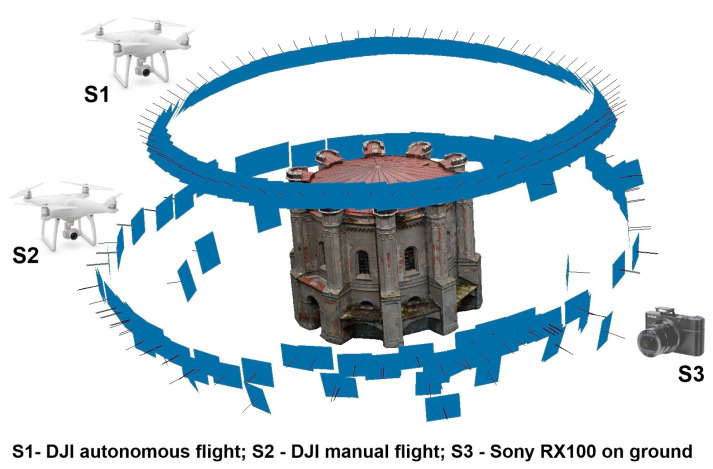
Photos taken using the DJI and Sony cameras.

To make a 3D model of a historical building we recommend two or three independent orbital flights around the object at different heights and with different camera angles of view to improve the quality of the final model (Figure 5).

For this reason and due to the trees in the immediate vicinity of the object and the buildings in the vicinity, the photographic documentation of the object was made in three stages. The first two stages of the inventory were made by a small DJI Phantom 4 PRO UAS (DJI, Shenzhen, China) flying at low altitude, but even before these stages, we started off by capturing nadir images. It outlined our modelling object and the area around it. Then we prepared a safe, autonomous flight plan for our UAV (first stage). Basic technical parameters of the quadcopter are presented in Table 1. 

In the first step, an autonomous flight was made in a circle around the upper, highest part of the water tower. The flight parameters were height 10 m, circle radius 15 m. The angle of the camera was approximately 45°. In the second step, to ensure the safety of the flying camera, the autonomous mission was abandoned. The trees are 410 m away from the tower and reach a height of 6 m. For this reason, in the central part of the object, the photos were taken in a manual mode, avoiding the obstacles (flight height 5 m). During this part of the mission the angle of the camera was close to 90°.

In the third stage, images have been acquired from ground around the building using an RX100 II camera (Sony, Tokyo, Japan). This camera is an element of the UAS fixed-wing equipment, which is mainly used for mapping larger areas [21]. The technical parameters of the DJI and Sony cameras are shown in Table 2.

### 3.2. TLS Data Acquisition

**Figure 6:**
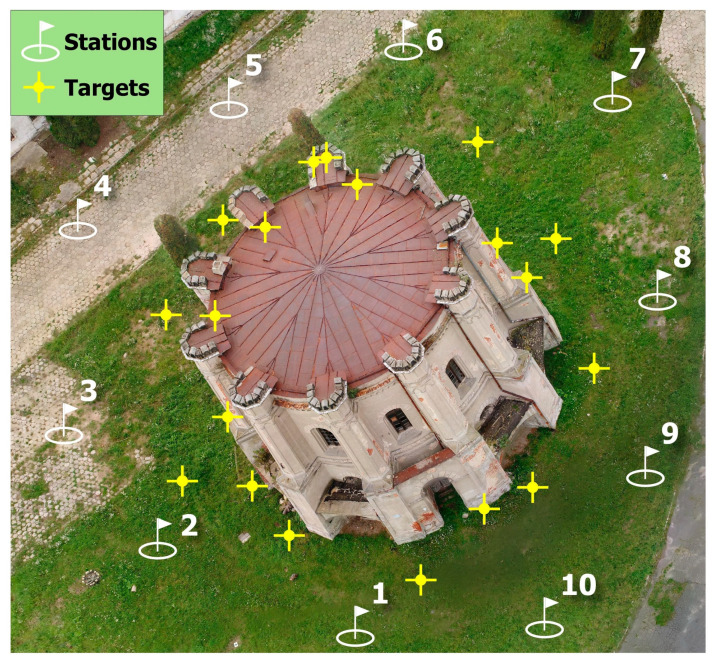
The locations of the TLS stations and the target points.

The TLS data was collected through a time-of-flight laser scanner (ScanStation C10, Leica Geosystems, Heerbrugg, Switzerland). Ten stations were used during field data collection (Figure 6). The technical parameters of Leica ScanStation C10 are presented in Table 3. Nineteen target spheres were evenly distributed around the building before scanning, and point clouds acquired from different stations were registered into the same coordinate system based on these target spheres.

The acquisition phase and the point cloud treatment was performed with laser scanner Leica ScanStation C10. During the acquisition, ten scan stations were arranged. The measurements were taken within one day (10 h). A high-quality digital camera integrated in the instrument was used to obtain photographic images and good textures for the model. During the measurement, the sky was cloudy, which guaranteed dispersed lighting and the shortest possible shadows.

The even distribution of target points and scanner stations around the building allowed us to obtain the full coverage of scans. Due to the height of the building and the location of the instrument at ground level, the obtained point cloud lacked data of the roof.

### 3.3. Point Cloud Comparison (UAV versus TLS)

**Figure 7:**
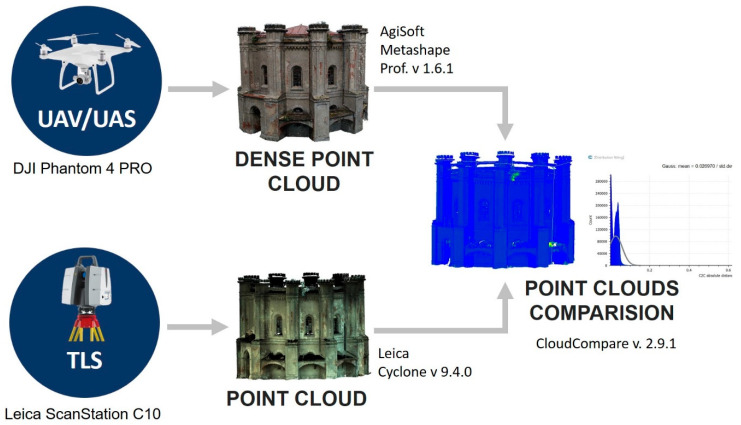
Methodology of UAV dense point cloud quality assessment.

The comparison between 3D models helps to detect changes in the building through the identification of redundant or missing elements in one model. This methodology also allows evaluating the usability of the new surveying methods by comparing the accuracy of the obtained results with the traditionally used methods. This section presents a methodology of UAV and TLS models change assessment. It was made by a comparison between point clouds of the Water Tower building collected by UAV and TLS sensors. The processing workflow is presented in Figure 7.

In order to assess the accuracy and effectiveness of the UAV acquisition method for smart tourism services, the analysis of the Water Tower building was conducted. The comparison of point clouds was performed with the use of the CloudCompare app (CloudCompare Development Team, 2019). Cloud Compare is an open-source software package designed specifically for point-cloud processing and contains significant mesh model/point cloud comparison tools.

In the first step, point clouds were registered into a common coordinate system. A coarse registration was done manually with CloudCompare software (v2.9.1, CloudCompare Development Team, 2019), by using three common points between them. The final root mean square error (RMS) of the alignment was 0.04 m.

Additionally, before the final comparison of the point clouds, they should be accurately registered as any misregistration error may be wrongly interpreted as a change in the environment. In the second step, a fine registration was made. It was done based on the Iterative Closest Point (ICP) algorithm by minimizing the point-to-point distances between the point clouds. The RMS for point cloud registered using the ICP was calculated as 0.14 m.

The differences between the UAV dense point cloud and the TLS survey were estimated by calculating the nearest neighbor point-to-point 3D distance for every point.

## 4. Results

The article presents the methodology of using UAV data for historical objects to prepare digital 3D assets for Web3D, AR/MR/VR smart cultural heritage services. The first step to prepare an object model for smart cultural heritage services is the process of data acquisition. The study describes a universal workflow on data acquisition using small UAVs, which can be replicated to support researchers, scientists, or practitioners in obtaining high accuracy 3D models for small historic buildings. The primary problem with the construction of smart tourism services is the lack of up-to-date, reliable 3D models. The presented solution shows how to obtain new 3D data very quickly. The proposed methodology can be used for tourism as well as for the inventory of historical objects for cultural heritage purposes. Special attention was given to planning the UAV measurements in order to obtain accurate, reliable source data. Finally, the quality and completeness of the obtained UAV point cloud was compared and evaluated on the basis of the point cloud from an independent surveying technique—TLS.

Data processing was divided into four phases:

- UAV point cloud processing,
- TLS data processing,
- UAV and TLS point cloud comparison,
- processing UAV data using 3D modeling software and creating texturing models of the building.

In the last stage, 3D models were tested for WebGIS, AR/MR purposes. The hardware configuration used for the tests was: OS Windows 10, 64 bit, CPU 2 x Intel(R) Xeon(R) CPU E52667– 3.30 GHz, RAM 64 GB, GPU(s) Quadro K2000, Hard Disk SSD 512 GB.

### 4.1. UAV Data Processing

The post-processing of UAVs images was carried out using the Metashape Prof. v 1.6.1 software (Agisoft, St. Petersburg, Russia). Only the images with the highest quality parameters were used for the orientation procedure of the photogrammetric datasets. In self-calibration mode, the dataset of the 223 images was processed. According to the photogrammetric pipeline, the data set was oriented in Metashape in line with the Self-Calibrating Bundle Adjustment. Later, the feature-based method was used. It does not require specific targets in the scene for photogrammetric acquisitions and makes a list of corresponding pairs by exploiting recognizable natural points and geometries as features. Additionally, scale bars were implemented based on the method presented by Cultural Heritage Imaging [49].

**Figure 8:**
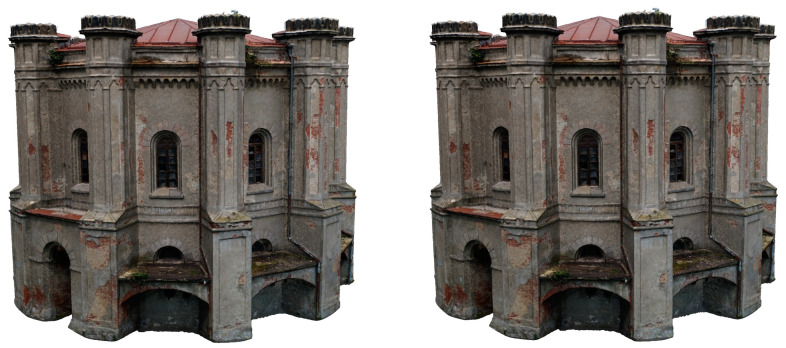
Visualization of UAV dense point cloud (**left**), texture shaded model (**right**).

To evaluate the quality of image matching (alignment), the number of the projections and the achieved error was taken into account. In the next step, an ultra-dense point cloud was built. In total, the final 3D point cloud was created, consisting of 50,288,792 points and textured mesh models (Figure 8).

### 4.2. TLS Point Cloud Processing

Postprocessing of the collected data was performed in Cyclone software version 9.4.0. An alignment of the acquired point clouds was initially performed based on the cloud-cloud approach, and then manually, by identifying control points between scans and using the ICP algorithm. Points outside the object of interest were removed from the point cloud. The final point cloud was saved in the American Society for Photogrammetry and Remote Sensing (ASPRS) LAS file format for further processing.

**Figure 9:**
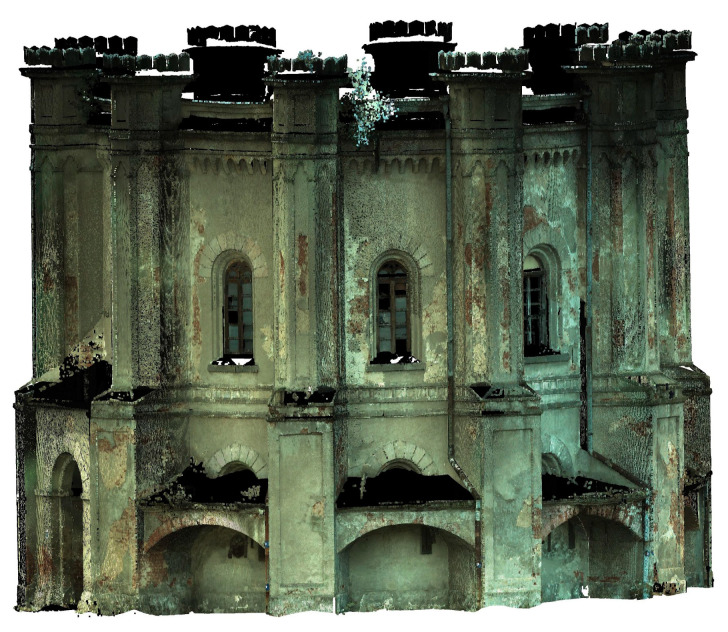
Visualization of the TLS point cloud.

As a result of the work carried out, a 3D model of the entire building was created, consisting of 41,356,675 points with 0.01 m RMS of alignment (Figure 9). The whole model was edited in successive phases of decimation, cleaning, and noise reduction.

### 4.3. UAV Point Clouds Accuracy Assessment

**Figure 10:**
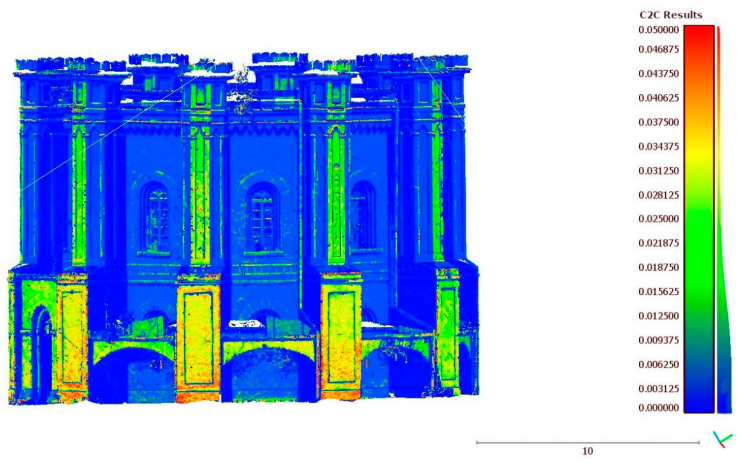

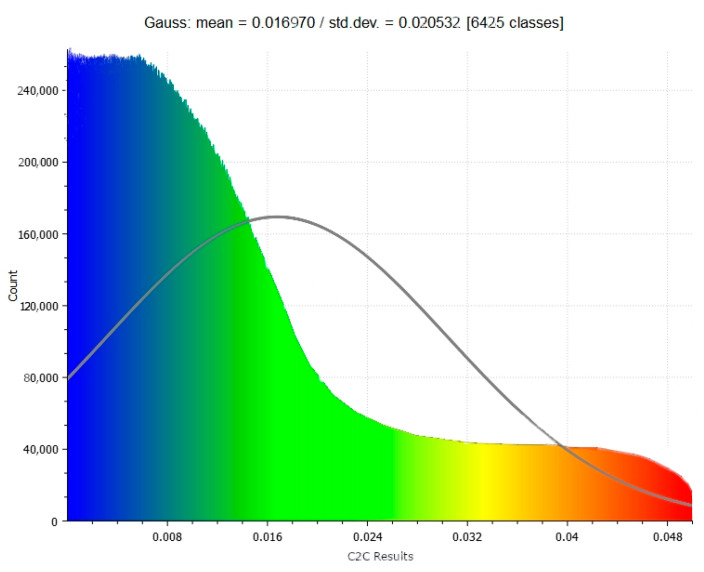
Cloud to cloud comparison (absolute distance calculation using Quadratic algorithm from CloudCompare).

The TLS comparison involved comparing the UAV point cloud with the point cloud obtained from the TLS. To assess the UAV model’s quality, the cloud-to-cloud (c2c) distance computation tool with a quadratic algorithm from CloudCompare software was used (Figure 7). As a result, CloudCompare software provides a color scale that shows the value of the c2c distance computation and statistical distribution of the difference values (as shown in Figure 10). The mean distance between point clouds was calculated at the level of 0.017 m, and standard deviation values at 0.021 m.

Figure 10 shows the range of changes between compared models. The differences obtained indicate that the compared point clouds are properly matched. Both TLS and UAV point clouds are very similar. The presented results confirmed the correctness of the UAV model. It was used in subsequent steps to create polygonal models of different complexity and then to test their suitability for WebGIS, AR/MR services.

### 4.4. Optimization of a Textured Model for AR/MR Purposes

Another important element of this study is to show the potential of the meshed model. Meshed models created on the basis of UAV data may include many millions of polygons, so it is practically impossible to directly render the full-size model in real-time without dedicating a hardware platform. This problem is particularly important for instruments with limited computing resources, like mobile devices (smartphones, tablets) and AR/MR (helmets, glasses). 

A raw 3D mesh typically has an enormous amount of detail and requires a simplification procedure. The ideal number of polygons depends on the application and the target platform. Mobile apps (Android/iOS) must have much simpler polygon models than web or desktop applications. 

This change in visual fidelity must be balanced when showing models on AR devices, VR helmets, or MR glasses such as HoloLens. These models should look as realistic and compelling as possible, while allowing these devices to operate efficiently. The modern UAV/TLS data processing software includes automated 3D model optimization tools. This functionality allows simplifying even complex models to fit within the available mobile or AR/MR device resources. A common rule is to balance the number of triangles in order to maintain the highest quality of objects. To create a model with proper mesh construction, a very high-density model is generated and the number of tris (triangles) in the model is reduced so that they run smoothly on their respective applications. 

The process of preparing 3D content for Web 3D/AR/MR/VR required model generalization and generation of additional 2D textures. There are numerous ways to achieve this goal. For our purpose, one general workflow of mesh and texture adaptation for Unity game engine was developed. In this section, the optimization process was described. It was conducted in several steps, which allowed the model to be simplified for multiple scenarios. At this stage, the models were re-processed, so less detail was needed to define them.

In the first stage, the specifications of AR/MR/VR devices (MS Hololens (Microsoft Corporation, Redmond, WA, USA), Oculus Rifts / Quests (Facebook, Inc., Menlo Park, CA, USA), HTC Vive (HTC Corporation, Xindian, New Taipei, Taiwan), Magic Leap (Magic Leap, Inc., Plantation, FL, USA) and related technical white papers and recommendations for the most famous Software Development Kits (SDKs) and Unity game engines were analyzed [50,51,52,53,54,55,56,57]. Technical feasibility and manufacturer recommendations for model optimization were analyzed. The following parameters were taken into account: rules for texturing, number of triangles, number of objects in the scene, suggested size and type of textures, scales, recommendations for model preparation.

The concept of building six models was considered—one reference model with the highest level of accuracy. Five derivative models were built on the basis of this model, 2 for desktop devices for VR, WebGIS, and 3 for mobile devices and network operation. After reviewing the documentation, we decided to adopt the following limits for the number of polygons (triangles)—10 M, 3.5 M, 1 M, 250 K, 100 K, 25 K (Table 4). The main goal was to reduce the impact of 3D objects on performance by adapting to the device performance and the selected software architecture (local/network). Firstly, the reference model, as the most accurate version of the model (high-quality model) was prepared. Secondly for scenarios with models stored locally on desktop devices and visualized directly on the client, medium and low-quality models were constructed. The last three models assume a network version with multiple users to share a model on a server for collaborative viewing.

**Figure 11:**
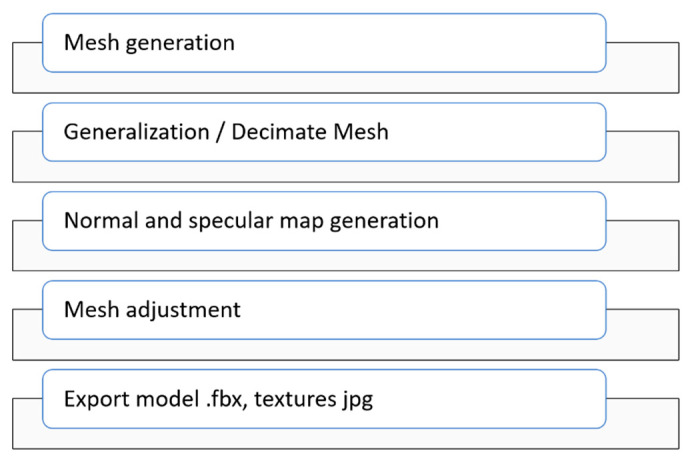
Methodology used for creating 3D models.

The preparation workflow for 3D models is presented in Figure 11. Initially, the reference model was generated using Agisoft Metachape software. In the following step, Metashape application was used to decimating the model in order to reduce the number of vertices of the model. According to the above assumptions, five models were created from the presented workflow. Based on high-poly model a Normal map was generated. Then the texture of the model was transferred from the high-poly model to the low-poly one. In the next step a diffuse map was generated using the previously created normal map. In the last step the mesh is generated and adjusted with the use of UV mapping mode. Finally, the mesh is imported into Blender in order to adjust its position and scale for use in the Unity game engine.

**Figure 12:**
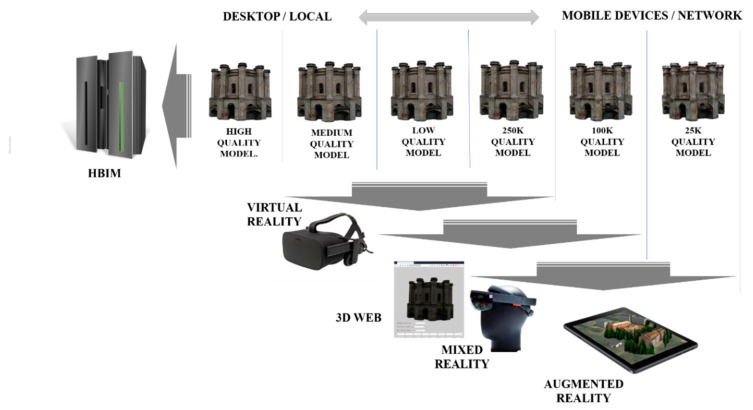
Visual illustration of the strategy of generating mesh models.

**Figure 13:**
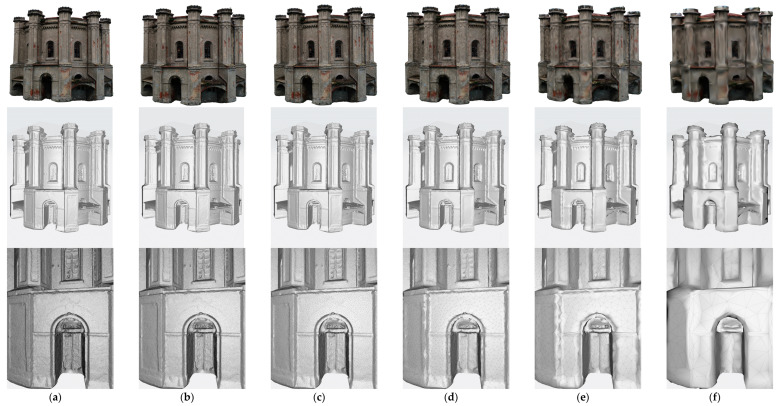
Comparison of mesh models of Water Tower building: (**a**) High quality model. (**b**) Medium quality model (**c**) Low quality model (**d**) 250 k quality model (**e**) 100 k quality model (**f**) 25 k quality model (top—models with textures, middle—solid model, bottom—models with triangles (bottom part of building with door)).

At this stage, six models were generated with various levels of detail/number of polygons (Figure 12). In the following, they have been tested for their usefulness for various visualization platforms WebGIS, AR/MR systems. The analysis was performed using a method that allows for comparing the visual quality of the optimized model with the reference model. In this way, the assumptions regarding the number of polygons and textures were verified by checking the performance on the devices on which Web 3D/AR/MR models were visualized (Figure 13).

### 4.5. Implementation of Proposed Methodology for Services using Web 3D /AR/MR Technology

**Figure 14:**
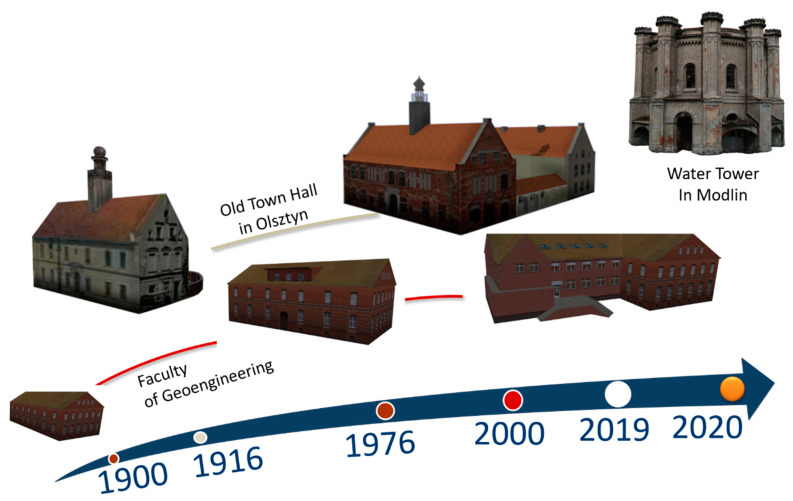
Concept of architecture that allow one to visualize temporary changes of the building façade based on the Water Town in Modlin (top), the Old Town Hall in Olsztyn (middle), main building of the Faculty of Geodesy, Geoengineering (bottom).

Smart cultural heritage services are based on the latest technical innovations. Recent technological breakthroughs in VR/MR/AR AR/MR/VR offer new functionalities for tourist attraction promotion. New technology provides an excellent opportunity to demonstrate the history of the building (changes and renovations over time). The concept of system architecture that allows to visualize temporary changes in the building is presented in Figure 14.

The presented solution assumes building models for different timeframes (database snapshots). The first option is the latest, most up-to-date model (As-is). Presented methodology based on the use of UAVs is one of the methods that allows to prepare an item presenting the current appearance of the building. Then the system database is extended with models showing the appearance of the historical building in the past. They start from the construction stage (As-build) through subsequent stages, including significant modifications (As-was-in-year).

The presented concept assumes visualization of changes in the building using various technical solutions, including 3D Websites, as well as dedicated mobile AR/MR applications. The unified development platform (Unity) allows to create several models dedicated to different platforms based on one reference model.

The concept of the system was presented based on three case studies. The first one presents a model of the water tower in Modlin. It contains only the latest model (As-is). The second is the main building of the Faculty of Geoengineering, which contains models from three phases (construction (As-build), first modernization (As-was), and the last phase showing the appearance after a major modernization (As-is). The third building is the Old Town Hall building in Olsztyn. It has two models; the first one reconstructed on the basis of historical documentation and original photographs of the facade from 1916, and the second one is a real model (created using UAS).

The following sections show the results of testing constructed 3D models. Five models have been tested with Web/AR/MR/VR applications. Finally, the possibilities of presenting temporary changes in the history of the building are also shown.

### 4.6. Testing Mesh Models Using 3D Web, MR and AR

The usability of particular mesh models has been tested in different environments. In the first stage, the models have been visualized using the methodology described by the authors in the article [17]. A web browser-based and 3D WebGIS solution were used, in which the models are visualized as Collada models using cartodb (https://carto.com/), jquery (https://jquery.com/) and three.js (https://threejs.org/) libraries. The proposed solution presents a thematic map with a layer of buildings in Olsztyn. Each object has attributes with information about the year of construction and recreation. The range of content displayed on the map is defined by the timeline slider. Only buildings whose year of construction is within the indicated time range are visible. 

**Figure 15:**
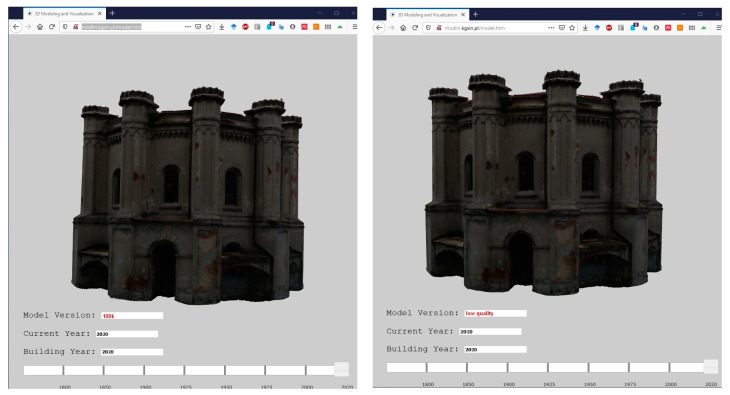
Visualization of the water station building model as a dae models in 3D web GIS environment.

The models were treated as one of the stages of building history and shown as the most recent stage of the building. The view of the selected models is shown in Figure 15.

In the next step, the ability to build a mobile AR application displaying a 3D model was tested. The application is based on Android Studio v. 3.2.1 and a cross-platform game engine Unity, developed by Unity Technologies. The tests implemented two technological approaches typically used in outdoor smart tourism services: markerless AR (location-based or sensor-based AR) and superimposition-based AR. Both provide excellent opportunities for the presentation of historical objects, and location-based technology allows the use of GNSS positions and presents AR content directly in the field. The application is based on the ARCore framework provided by Google. Two main functionalities offered by ARCore are: tracking the position of a mobile device while it is moving and building its understanding of the real world.

**Figure 16:**
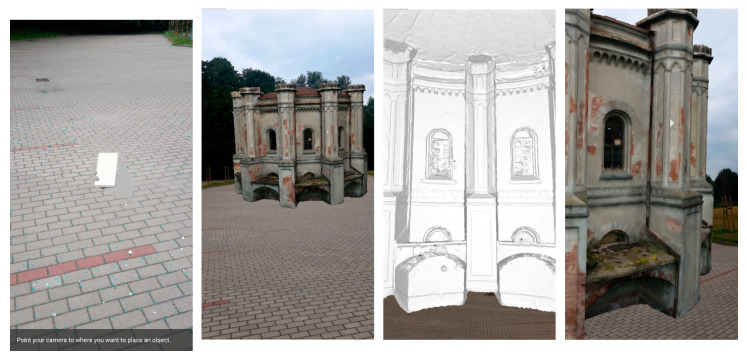
Visualization of the 3D water station building model in prototype AR application—100 k model (from the left surface recognizing, walking near 3D model of Water Station (real sizes), view from inside the building, view from near the building). Test carried out in the parking lot in Olsztyn on the UWM campus.

In Figure 16 screenshots from the smartphone are presented. They show the appearance of superimposition AR content (100 k building model) on the physical world observed by the camera, contained in a smartphone. In order to display the model, it is necessary to recognize the surface (paving block in the figure) and then indicate the point at which the model is displayed. The figure shows the model in real size. It is possible to move around the building, as well as enter its interior.

**Figure 17:**
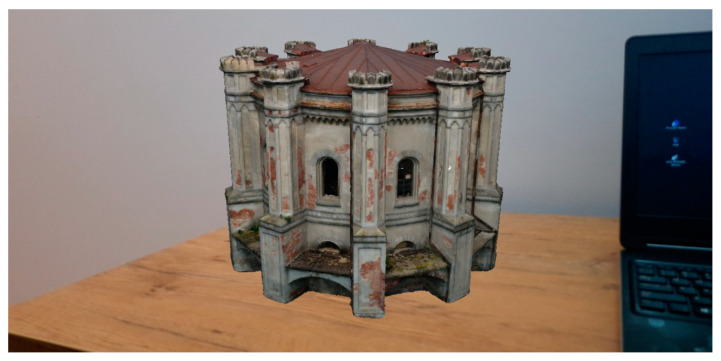
Visualization of the 3D water station building model in prototype AR application superimposed on the desk (100 k model).

In the next stage the models were tested and visualised on the desk (Figure 17). The application enables to present the models on a scale, thanks to which it is possible to analyse the quality of the model. It is also possible to scale, rotate and move the model. Switching on and off layers.

**Figure 18:**
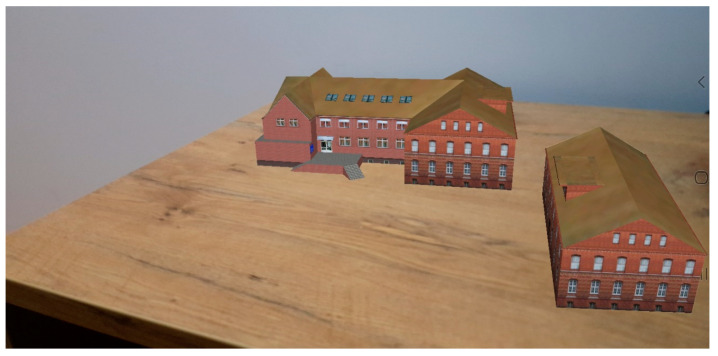
Visualization of temporal changes of the Dean’s Office of the Faculty of Geoengineering building (at the top 3D model of current look of the building, on the right model from 1976).

The AR application can visualize multiple models at the same time (Figure 18). This method was used to display two models of the same building to show its appearance in 1976 and 2002. The application offers additional functionalities, such as zooming, moving, and rotating the models. The gesture-based functionality helps to analyse existing differences and evaluate changes. The comparison shows that in 2002 a new part was added to the historic part of the building. A new entrance was also added, but the appearance of the building was maintained in the same style.

**Figure 19:**
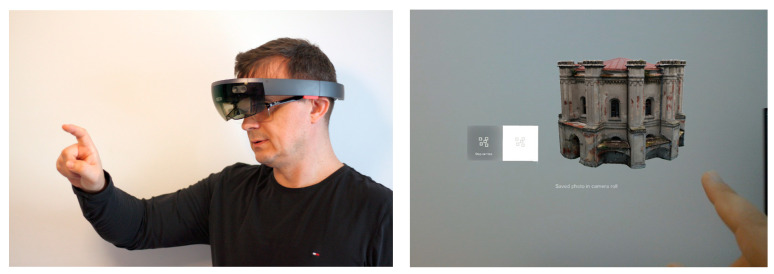
Visualization of the water station building model as a hologram using Microsoft Hololens MR helmet.

To check models in the MR environment the Microsoft Hololens helmet (Microsoft Corporation, Redmond, WA, USA) was used. A test application has been created that displays the water station building as a hologram and enables the user to interact with it using gestures. The application was made using Microsoft Visual Studio IDE (Integrated Development Environment) and a cross-platform game engine Unity, developed by Unity Technologies. It provides an interface that allows you to rotate, move, and scale tested models using gestures or Clicker hardware. The process is presented in Figure 19.

## 5. Discussion and Conclusions

The smart city offers a comprehensive range of activities for the development of urban agglomerations and local communities. One of the most important aspects influencing community development is building the identity of the inhabitants through cultural evolution and access to cultural heritage. These opportunities are provided by introducing new smart tourism services that promote local culture, tradition, and history. 

The acquisition of spatial data and further modeling is a long-term, cost-intensive process. Historical objects require involvement of specialists from various disciplines. The interdisciplinary character of the tasks means that data acquisition is based on the HBIM methodology. This methodology renders results in the form of advanced 3D proof-reading of HBIM models, including databases containing data describing each object. However, it results in the limited number of such models and their dispersed locations. There are no available standards of workflow, and there are no open repositories giving access to historical 3D models.

New measurement techniques, especially photogrammetric methods, provide the opportunity for a rapid acquisition of reliable data. The article presents a methodology that demonstrates the possibilities of using low-cost photogrammetry and UAVs as a measurement platform allowing for a quick data collection for smart tourism services. As a test area, one of the objects which is a part of the largest 19t h-century fortresses in Poland was chosen. The 3D building model was prepared and optimized. Additionally, the possibility of combining UAV products with existing datasets and models, different characteristics in terms of construction methodology, accuracy, and reliability was tested.

The study has shown that low-cost UAV/UAS with non-metric cameras can be an interesting alternative to the already used methods. They allow to produce 3D models of historical buildings with high accuracy, that can be almost directly used in cultural heritage services and LBS applications. The most important advantages of the UAS based photogrammetry are: a cost-effective and quick way of accurate 3D scanning, measurement, surveying, and reality capture in the documentation of the cultural heritage. In the proposed approach, we suggest unstructured geometric reconstruction of the building outer surface based on point cloud. The geometric accuracies of the obtained models are 2–3 cm. This accuracy level allows us to use it also for the conservation and/or monitoring of archaeological heritage or architectural restoration purposes. The achievable results could even be better if a higher resolution professional camera were used.

Virtual and augmented reality technology is becoming increasingly popular. Virtual reality (VR), augmented reality (AR), and mixed reality (MR) are changing the way people perceive the digital world. Gartner indicates that by 2028, users’ experiences will change significantly in the way they perceive the digital world and interact with it [58]. He claims that perception and interaction models will move to multi-sensors and multimodal experience. Many researchers are currently working on the influence that recent technological breakthroughs in VR/MR/AR have on user experience. Interesting works were presented by Flavian [59], Griffin [60], Yung [61].

Most of the current research on tourism and historical sites is focused on the use of virtual reality in museums [62]. The implemented solutions usually provide extended opportunities of presenting selected objects or exhibitions. They increase the level of immersion by moving the user to the virtual world as a whole [63].

AR technology brings new possibilities for content presentation. It enables adding content to the real world observed by the user. This technology allows us to visualize, even in natural dimensions, 3D models of objects located in distant locations. It improves the perception of the observed objects by superimposition of computer-generated content, descriptions, sounds, videos, and animations. There are two technological approaches typically used in outdoor smart tourism services: markerless AR (location based or sensor-based AR) and superimposition-based AR. Both provide excellent opportunities to present historical objects, while location-based technology allows adding content to a physical object. Apart from visualizing the objects, it is also possible to visualize temporal changes in buildings.

In addition, it is possible to add a lector’s voice and to present selected parts of objects. This technology can also be widely used by adding historical animations to outdoor objects, such as open-air museums [64]. The only limitation is devices (smartphone/tablet). Their technical parameters are a significant limitation in presenting advanced 3D models or animations. Thus, optimization of digital AR content is becoming an essential factor.

MR is the most advanced visualization method. It enhances the level of immersion thanks to goggles, which not only offer the possibility to visualize digital content/3D models but also to interact with these objects through touch and gestures. The MR application offers the highest level of immersion and the best capabilities to interact with virtual objects.

The presented AR/MR methods are of particular importance at the time of the global health crisis caused by the coronavirus pandemic COVID-19. This has a significant impact on the tourism industry due to the resulting travel restrictions, as well as a decrease in demand among travelers. Virtual tourism based on AR/MR can be a breakthrough solution that will change the limitations of access to historical objects, enabling dynamic development of smart cultural heritage services.

The research has indicated several problems in the implementation of different models for tourism services based on AR/MR or web. The use of UAVs for data acquisition allows to work with models with real dimensions and to maintain the scale of objects. The model’s scale is correct and based on the vertices, so the normal model transformation matrix has no scaling (1 Unity unit = 1 m).

Recommended number of polygons depends mostly on the software and hardware. In our opinion, the optimal number ranges between 25,000 and 50,000 for mobile devices. For web apps, this number should be less than 250,000, and for desktop apps up to 2,500,000 for the whole scene. The number of calls drawn is often more limiting than the total number of polygons. It is beneficial to present one or more complex meshes than too many separate, less complicated models.

Based on the analysis of the use of mesh models for WebGIS, AR/MR, it was found that it is a good practice to make multiple Level of Detail models. This makes it possible to switch between different level of detail (LOD) models as users move away from the model. It prevents overloading of used devices, while maintaining a good framerate.

The proposed methodology provides a great opportunity to create solutions at the local level. It allows for the promotion of interesting local sites and historical monuments. It also enables the development of mobile applications that meet young generation requirements. It changes classical storytelling methods and combines them with new technology, especially data visualization methods (3D Web/AR/MR). It reviewed the digital documentation of heritage using low-cost UAV, TLS, LIDAR 3D parametric HBIM models of heritage buildings. The proposed framework was implemented in the Modlin water tower, located near a unique military complex near the Vistula and Narew rivers in Poland. The procedure for heritage digital documentation consists of three tiers: (1) Collecting Raw data using UAV and TLS, (2) Process acquired data and prepare point clouds, and (3) Generate the parametric model. AgiSoft Metashape was used to help in tracing the as-built point cloud data captured by the laser scanner. The workflow provides new tools and methods for 3D virtual heritage modeling, documentation, management, and analysis.

The potential intersections of AR/MR and smart environments are yet to be explored further. The conducted research is a part of the work related to the use of crowdsourcing and volunteered geographic information methodology in the process of obtaining data on historical objects. It will combine the capabilities of GIS systems and HBIM databases to create a solution for the visualization of temporal changes in the look of historical buildings.

## Figures and Tables

**Table 1:** Technical specification of DJI Phantom 4 Pro UAS.

Characteristics	UAS Parameters
UAS model	DJI Phantom 4 PRO
UAV type	Quadcopter
Total mass	1.388 kg
Diagonal size	350 mm
Max wind speed resistance	10 m/s
Battery	Intelligent Flight Battery LiPo 4S 5870 mAh
Max flight time (in practice)	25 min
Camera	DJI 1” CMOS 20M
Satellite positioning system	GPS/GLONASS
Max tilt angle	42°
Radio control	2.4/5.8 Hz

**Table 2:** DJI and Sony RX100 II cameras technical specification.

	Camera	Phantom 4 PRO	RX100 II
Parameters			
**Manufacturer**		DJI	Sony Exmor R
**Sensor**		1” CMOS	1” CMOS
**Effective Pixels**		20M	20.2M
**Lens**		FOV 84° 8.8/24 mm F/2.8−F/11	Carl Zeiss Vario-Sonnar T 10.4–37.1 mm F/1.8−F/4.9
**Photo**		JPG, DNG (RAW), JPG+DNG	JPG, Sony ARW 2.3 (RAW), RAW+JPG
**Operating Temperature**		0 ℃−40 ℃	0 ℃−40 ℃
**Max Image Size**		5472 × 3648 (3:2) 4864 × 3648 (4:3) 5472 × 3078 (16:9)	5472 × 3648 (20.0 MP, 3:2) 4864 × 3648 (17.7 MP, 4:3) 5472 × 3080 (16.9 MP, 16:9)
**Recording Media**		MicroSDHC, MicroSDXC	MS Duo, MS PRO Duo, SD, SDHC, SDXC
**Stabilization**		3-axis (pitch, roll, yaw)	n.a.

**Table 3:** Leica ScanStation C10 technical parameters.

Parameters	Leica ScanStation C10
Ranging Method	Time of flight
Angle Precision	
Horizontal/Vertical	12’’/12’’
Modeled Surface Precision	2 mm
Range	30 m ≅ 90%; 134 m ≅ 18%
Minimal Step of Scanning	1 mm
Scan Rate	50,000/sec.
Laser Class	#R, green (ƛ = 532 nm)
Spot Size	0−50 m ≃ 4.5 mm
Field of View	
Vertical/Horizontal	270°/360°

**Table 4:** Main characteristics of the 3D models.

**Model 1 (high quality model)**	Faces: 10,000,000 Vertices: 5,033,189
**Model 2 (medium quality model)**	Faces: 3,500,000 Vertices: 1,679,712
**Model 3 (low quality model)**	Faces: 1,000,000 Vertices: 511,101
**Model 4 (250 K quality model)**	Faces: 250,000 Vertices: 125,728
**Model 5 (100 K quality model)**	Faces: 100,000 Vertices: 50,141
**Model 6 (25 K quality model)**	Faces: 25,000 Vertices: 12,014
